# Mobilization exercises in preparation to bracing must be only at start of brace wearing. Results from a prospective controlled study

**DOI:** 10.1186/1748-7161-7-S1-O49

**Published:** 2012-01-27

**Authors:** M Romano, A Negrini, S Parzini, S Donzelli, F Zaina, S Negrini

**Affiliations:** 1 ISICO, Milan, Italy

## Purpose/background

Some exercises protocols (SEAS, Lyon) for scoliosis patients before brace wearing require a period of mobilization exercises to reduce spine and muscle stiffness, and obtain a better action of the brace [1,2]. Aim of this study is to define when these exercises should be applied to achieve the best results of bracing.

## Materials and methods

Population: 357 consecutive scoliosis patients with 23 hours per day brace prescription (299 females, Age 13.2±11.1 °Cobb: 41.4±9.5, Risser 1.2±1.2). Control: out-of-brace 6 months x-ray. The 206 SEAS patients have been divided according to time elapsed between start of mobilization exercises and start of brace wearing. We also had two control groups: one Usual Physiotherapy (UP: 115) and one No exercises (NE: 36) Statistical analysis: Anova, T-Test and Relative Risk.

## Results

At baseline there were some differences, with the SEAS patients worst than UP and NE. All patients in all groups improved in almost all parameters with brace treatment (more in SEAS than UP and NE). Best results (statistically significant for Cobb degrees, ATR and Trace in comparison with other SEAS groups, but also with UP and NE) have been obtained by patients who performed mobilizing exercises almost at the same time with start of bracing.

## Conclusions

Despite our starting idea, that spinal mobilization exercises should start at least two months before bracing, our results show that they are more effective when patients perform this protocol at the same time in which they start wearing the brace. This drove to change SEAS protocol accordingly.
